# Single Extracellular Vesicle Analysis Using Flow Cytometry for Neurological Disorder Biomarkers

**DOI:** 10.3389/fnint.2022.879832

**Published:** 2022-05-17

**Authors:** Houda Yasmine Ali Moussa, Nimshitha Manaph, Gowher Ali, Selma Maacha, Kyung Chul Shin, Samia M. Ltaief, Vijay Gupta, Yongfeng Tong, Janarthanan Ponraj, Salam Salloum-Asfar, Said Mansour, Fouad A. Al-Shaban, Hyung-Goo Kim, Lawrence W. Stanton, Jean-Charles Grivel, Sara A. Abdulla, Abeer R. Al-Shammari, Yongsoo Park

**Affiliations:** ^1^Neurological Disorders Research Center, Qatar Biomedical Research Institute (QBRI), Hamad Bin Khalifa University (HBKU), Qatar Foundation, Doha, Qatar; ^2^Deep Phenotyping Core, Research Branch, Sidra Medicine, Doha, Qatar; ^3^Qatar Environment and Energy Research Institute (QEERI), Hamad Bin Khalifa University (HBKU), Doha, Qatar; ^4^College of Health and Life Sciences (CHLS), Hamad Bin Khalifa University (HBKU), Qatar Foundation, Doha, Qatar

**Keywords:** exosome, extracellular vesicle, biomarker, NCAM, neurological disorder

## Abstract

Extracellular vesicles (EVs) are membrane vesicles released from cells to the extracellular space, involved in cell-to-cell communication by the horizontal transfer of biomolecules such as proteins and RNA. Because EVs can cross the blood-brain barrier (BBB), circulating through the bloodstream and reflecting the cell of origin in terms of disease prognosis and severity, the contents of plasma EVs provide non-invasive biomarkers for neurological disorders. However, neuronal EV markers in blood plasma remain unclear. EVs are very heterogeneous in size and contents, thus bulk analyses of heterogeneous plasma EVs using Western blot and ELISA have limited utility. In this study, using flow cytometry to analyze individual neuronal EVs, we show that our plasma EVs isolated by size exclusion chromatography are mainly CD63-positive exosomes of endosomal origin. As a neuronal EV marker, neural cell adhesion molecule (NCAM) is highly enriched in EVs released from induced pluripotent stem cells (iPSCs)-derived cortical neurons and brain organoids. We identified the subpopulations of plasma EVs that contain NCAM using flow cytometry-based individual EV analysis. Our results suggest that plasma NCAM-positive neuronal EVs can be used to discover biomarkers for neurological disorders.

## Introduction

Extracellular vesicles (EVs) are lipid bilayer-enclosed vesicles that exist in all body fluids including blood. EVs comprise exosomes and ectosomes, which are distinguished by biogenesis, content, size, release pathways, and function (Kalluri and LeBleu, 2020). Exosomes with the size range of 50–150 nm in diameter originate from the endosomal pathway and are released by the membrane fusion of multivesicular bodies (MVBs) with the plasma membrane, whereas ectosomes ranging from 50 nm to 1 μm in diameter are secreted through the plasma membrane budding (Kalluri and LeBleu, 2020). EVs are secreted from cells and involved in cell–cell communication by the horizontal transfer of the EV contents (Veziroglu and Mias, 2020).

Extracellular vesicles play critical roles in health and disease and have a potential clinical utility as novel biomarkers for early diagnosis and therapeutic targets for treatment (Trino et al., 2021). Given that EVs reflect the cell and tissue of origin in terms of disease prognosis and severity, the contents of EVs provide non-invasive biomarkers for several diseases (Trino et al., 2021). Brain-derived EVs might provide biomarkers for neuronal disorders, and EVs can be used in therapeutics as a drug delivery system to the brain (Mustapic et al., 2017; Yoo et al., 2018). EV proteins and RNA are considered promising biomarkers for neurodegenerative disease and neurodevelopmental disorders (Guix et al., 2018; Pulliam et al., 2019; Saeedi et al., 2019).

Due to the limited accessibility to the brain and cerebrospinal fluid (CSF) for biomarker discovery, blood is ideal for liquid biopsy, given its easier accessibility and non-invasive collection (Marrugo-Ramirez et al., 2018). Intriguingly, EVs can cross the blood-brain barrier (BBB) (Alvarez-Erviti et al., 2011; Chen et al., 2016; Saeedi et al., 2019), circulating through the bloodstream. Thus, plasma EVs provide a potential therapeutic approach to neurological disorders. However, neuronal EV markers in blood plasma remain unclear. EVs are so heterogeneous in size and contents that bulk analyses of heterogeneous EVs using Western blot and ELISA might have limited utility, and analysis of individual neuronal EVs isolated from plasma using flow cytometry has been challenging.

In the this study, we used a BD FACSAria III flow cytometer to investigate individual plasma EVs isolated without using beads. EVs were isolated from the plasma of healthy donors using size exclusion chromatography (SEC), removing plasma proteins. Around 95% of EV samples were positive for CD63, a marker of exosomes, strongly suggesting that our EV samples are mostly exosomes (Mathieu et al., 2021). Neural cell adhesion molecule (NCAM) is highly expressed and enriched in EVs released from induced pluripotent stem cells (iPSCs)-derived cortical neurons. Subpopulations of blood plasma exosomes contain NCAM, suggesting its origin from neuronal cells. Taken together, our data propose that NCAM-positive neuronal exosomes isolated from blood plasma can be detected and analyzed using flow cytometry. Neuronal EVs identified here will pave the way for further studies to discover biomarkers for neurological disorders.

## Materials and Methods

### Blood Collection and Plasma Preparation

The studies involving human participants were reviewed and approved by the Institutional Review Board (IRB# 2018-024) of Qatar Biomedical Research Institute (QBRI). Human peripheral blood samples were drawn from healthy donors into EDTA tubes (366643, BD diagnostics). In the processing of blood samples, blood components were separated using density gradient centrifugation. Briefly, blood samples were slowly layered over Histopaque-1077 (10771, Sigma), at a 1:1 ratio, and centrifuged at 400 *g* for 30 min with acceleration three and without brake at room temperature (Dhurat and Sukesh, 2014). After centrifugation, the plasma collected in the upper fraction was carefully removed into a new tube, avoiding the peripheral blood mononuclear cell layer below. The plasma supernatant was further centrifuged at 1,811 *g* for 15 min to remove platelets and blood cells to obtain platelet-free plasma, which was aliquoted and stored at −80°C until further use for EV isolation.

### Neuronal Differentiation and Brain Organoid

Neural precursor cells (NPCs) were generated from the human pluripotent stem cells through dual SMAD inhibition using SB431542 (S4317, Sigma) and dorsomorphin (ab120843, Abcam) as reported in previous studies (Khattak et al., 2015; Kutsche et al., 2018). Cryopreserved NPCs were thawed and cultured in six well-plate coated with 0.1 mg/ml poly ornithine and 20 μg/ml laminin for at least 3–5 days or until cell confluency was reached. The NPCs culture media contained DMEM F12 (11320-074, Thermo Fisher Scientific) with added 1% N2 (17502048, Thermo Fisher Scientific), 2% B27 (12587-010, Thermo Fisher Scientific), 1 μg/ml Laminin (23017015, Thermo Fisher Scientific), 2 μg/ml Heparin (H3149, Sigma), 1% non-essential amino acids (11140-050, Thermo Fisher Scientific), 10 ng/ml basic fibroblast growth factor (13256-029, Thermo Fisher Scientific), and 1% antibiotics (15140122, Thermo Fisher Scientific). For neuronal differentiation, NPCs were detached using accutase, and a cell density of 50,000 cells were seeded into 24 well-plate, coated with 0.1 mg/ml poly ornithine and 20 μg/ml laminin. The neuronal differentiation media contained freshly prepared 10 ng/ml glial-derived neurotrophic factor (GDNF) (450-10, Peprotech), 10 ng/ml brain-derived neurotrophic factor (BDNF) (450-02, Peprotech), 10 ng/ml insulin-like growth factor 1 (IGF-1) (AF-100-11, Peprotech), 10 mM dibutyryl-cAMP (1141, Tocris), 200 μg/ml ascorbic acid (A4403, Sigma), 1% N2, 2% B27, and 1% antibiotics in neurobasal media (21103-049, Thermo Fisher Scientific). Cells were maintained in a CO_2_ incubator for 35–40 days with differentiation media changed every second day. The cell culture media were collected for EV isolation and analysis.

The iPSCs were differentiated into cortical organoids based on published protocols (Qian et al., 2016; Sloan et al., 2018). Briefly, the iPSCs derived embryoid bodies (EBs) were differentiated in EB medium (DMEM/F12, 15% knockout serum replacement medium), 1× non-essential amino acids, 1× L-glutamine, 50 μM 2-mercaptoethanol, 0.2× penicillin/streptomycin) supplemented with 10 μM SB431542 (Stemgent), and 2 μM dorsomorphin (Sigma). On day 8, the organoids were embedded in Matrigel (BD Biosciences) and cultured in cortical organoid medium (1:1 DMEM/F12, neurobasal, 1× B27, 1× non-essential amino acids, 1× L-glutamine, 50 μM 2-mercaptoethanol, and 0.2× penicillin/streptomycin. On day 14, the organoids were mechanically dissociated from matrigel and cultured in the cortical organoid medium supplemented with 20 ng/ml EGF and 20 ng/ml bFGF, 2 μg/ml heparin, and 2 μg/ml insulin for 10 days. At day 25, EGF and bFGF were withdrawn and the organoids were cultured in cortical organoid medium supplemented with 20 ng/ml BDNF and 20 ng/ml GDNF. From day 43 onward the organoids were cultured in a maturation medium consisting of neurobasal (Gibco), 1× B27 (Gibco), 1× non-essential amino acids, 1× L-glutamine, 50 μM 2-mercaptoethanol, and 0.2× penicillin/streptomycin.

### Extracellular Vesicles Isolation

Total EVs were isolated from 0.25 ml of plasma by SEC using qEV2/35 nm SEC columns (SP8, Izon Science, Christchurch, New Zealand) following the manufacturer’s operating instructions with minor modifications. Briefly, plasma was thawed on ice and diluted with at least double the volume of phosphate-buffered saline (PBS). To remove cellular debris, the diluted plasma was centrifuged at 3,000 *g* for 10 min (4°C). The supernatant was collected and transferred to a new microcentrifuge tube, then centrifuged at 10,000 *g* for 30 min (4°C) to pellet down large vesicles. SEC columns were equilibrated to room temperature 1 day prior to use and flushed with 100 ml of freshly filtered PBS. The plasma sample was then added to the loading reservoir of the column and allowed to run through before adding PBS. The first 14 ml of void volume was discarded, and the following 6 ml of EV-zone fractions were collected separately or pooled depending on the downstream experiments. The amount of EV proteins was estimated by measuring the absorbance at 280 nm (A280). We collected fractions 1–5 from SEC and combined them as EV samples and confirmed that most of the plasma proteins are removed from the fraction of EVs. The pooled EV fractions were concentrated using pre-conditioned 100 kDa Amicon Ultra-15 centrifuge filters (UFC9100, Millipore) to a final volume of 500 μl. EV samples were aliquoted to minimize the freeze-thaw cycles and stored at −80°C until further analyzed.

### Nanoparticle Tracking Analysis

Particle size and concentration were determined using nanoparticle tracking analysis (NTA) (ZetaView, Particle Metrix, Germany). EV samples were diluted with filtered PBS to an average of 80 particles per frame and a final volume of 1 ml. Zetaview software (version 8.04.02 SP2) recorded particles at 11 camera positions and 30 frames per second.

### Atomic Force Microscopy

A drop of EVs was deposited over poly-D-lysine coated glass coverslips and incubated at RT. After 1-h incubation, the coverslip was washed twice with ddH_2_O and air-dried before imaging. AFM imaging was conducted on the Bruker Dimension Icon platform (Bruker, United States). A Scanasyst mode (with a scanasyst-air probe) based on the peak force tapping technique is selected for morphological analysis. All measurement was conducted in air at room temperature. The data analyses were performed on nanoscope analysis 2.0.

### Transmission Electron Microscopy

Extracellular vesicle samples were prepared adapting a previously published protocol (Thery et al., 2006), with some modifications. Briefly, 5 μl of sample fixed with 2% paraformaldehyde were deposited on carbon-coated 400-mesh copper grids (CF400-CU, Electron Microscopy Sciences). Grids were stained with uranyl acetate for negative staining and embedded in methylcellulose-uranyl acetate. EVs were visualized at 80 kV in Talos F200C Transmission Electron Microscope (Thermo Fisher Scientific). The images were acquired using bottom-mounted CETA camera.

### Western Blot

Western blotting was done to test for EV protein and non-EV protein markers. Equivalent EV particle numbers were dissolved under reducing or non-reducing conditions and heated at 95°C for 5 min. EVs were loaded on 4–20% Mini-PROTEAN TGX Precast Protein Gels (Bio-Rad), and proteins were transferred to nitrocellulose membrane (88018, Thermo Fisher Scientific). Blots were then blocked with 5% skim milk in TBST for at least 1 h at room temperature. Immunoblotting was done overnight at 4°C with the following antibodies at the appropriate dilutions: CD63 antibody (NBP2-42225, Novus Biologicals, 1:1,000), albumin antibody (ab10241, Abcam, 1:1,000), ApoA1 antibody (MIA1405, Thermo Fisher Scientific, 1:2,000), and NCAM antibody (Sc-106, Santa Cruz Biotechnologies, 1:500). The blots were washed the next day and incubated with goat anti-mouse-HRP secondary antibody (G21040, Thermo Fisher Scientific, 1:20,000). The protein bands were subsequently scanned using the ChemiDoc imaging system (BioRad).

### Flow Cytometry

The flow cytometry data were obtained using a BD FACSAria III SORP cell sorter (Beckton Dickinson). EV samples were diluted 20-fold in filtered PBS and stained with either DiD or DiI lipophilic dyes (V22889, Thermo Fisher Scientific), CD63-PE-CY7 antibody (25-0639-42, Thermo Fisher Scientific), or NCAM-FITC antibody (67184, cell signaling technology). To decrease non-specific positive events due to aggregates, lipophilic dyes and antibodies were subjected to high-speed centrifugation using Airfuge (Beckman Coulter). Excitation for scatter (FSC and SSC) was by 488 nm (blue) laser and excitation for Dil dye by 561 nm (yellow-green) laser. Dil emission was recorded at 585/15 nm. Settings were optimized for small particle detection. The lowest flow rate (1 μl/min) was applied for all measurements. The acquisition time was held constant for all samples and a log scale was applied throughout. The following controls were included to test for non-specific binding and autofluorescence: EV-only, antibody-only, and buffer-only. All samples were incubated simultaneously for 30 min at room temperature. Then, samples were further diluted in filtered PBS and analyzed using the BD FACS Aria III flow cytometer. Samples were run at 1 ml/min for 30 s. Graphs were created using GraphPad Prism version 9.0 (GraphPad Software). Flow cytometry analysis was done using FlowJo version 10.8.0.

### Immunostaining

Human iPSC-derived cortical neurons grown on coverslip were fixed using 4% paraformaldehyde solution (pH 7.4) for 8–10 min at room temperature. Samples were then washed using phosphate buffered saline (PBS) and plunged into 50 mM glycine solution for 15 min to avoid unnecessary antibody binding. Then, the samples were washed in PBS and blocked using 0.1% Triton X-100 and 10% donkey serum in PBS for 1 h. Primary antibodies MAP2 (NB100-1028S, Novus Biologicals LLC) and NeuN (702022, Thermofisher Scientific) were diluted in the aforementioned blocking solution and incubated overnight at 4°C. After the incubation, neurons were washed three times with PBS containing 0.1% Tween-20 (PBST) for 15–20 min. Secondary antibodies (715-545-150, Jacksons Laboratories; A10042, Thermo Fisher Scientific) were incubated for 1 h at dark and washed using 1× PBST. Coverslips were mounted using the antifade medium containing the nuclear stain (P36941, Thermo Fisher Scientific), sealed, and observed using Zeiss LSM Confocal microscope at ×63 magnifications.

The cortical brain organoids were fixed in 4% paraformaldehyde (PFA) for 3–4 h at room temperature. Organoids were washed with PBS and incubated in 30% sucrose solution overnight. Subsequently, they were transferred into an embedding medium (Tissue-Tek OCT compound, Sakura Finetek), snap-frozen on dry ice, and sectioned 16 μm with a cryostat (Leica). For immunostaining, the freezing medium was washed with PBS, and tissues were permeabilized with 0.3% Triton-X in PBS for 40 min. Tissues were blocked in 3% BSA in PBS containing 0.2% Tween-20 (PBST) for 2 h. Primary antibodies BRN2 (1:200, cell signaling, 12137), CTIP2 (1:200, cell signaling, 12120), and SOX2 (1:300, Invitrogen, MA1-014) were diluted in blocking solution and added to the tissue overnight at 4°C. The secondary antibodies were diluted in blocking solution and added to the tissues for 2 h at room temperature. Nuclei were stained with Hoechst33258 (Life Technologies) and tissues were mounted for microscopy on glass slides using Aquamount (Thermo Scientific). Images were captured by using an inverted fluorescence microscope (Olympus IX 53) and processed in ImageJ (Fiji).

## Results

### Enrichment of Extracellular Vesicles Using Size Exclusion Chromatography

**FIGURE 1:**
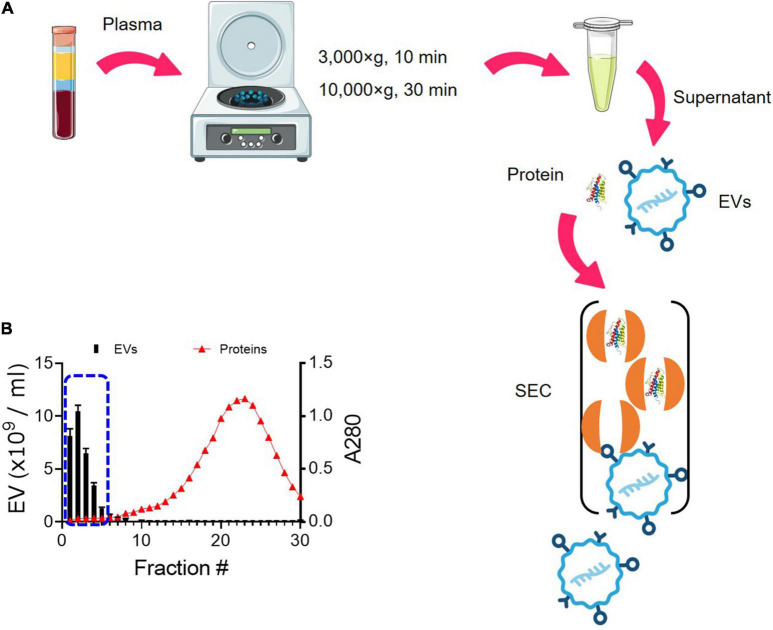
Enrichment of EVs from plasma using SEC. **(A)** Schematic overview of the procedure for isolating EVs from plasma using SEC. EVs elute first, followed by smaller protein complexes. **(B)** Elution profiles of EVs and plasma protein. EV particle numbers and protein concentration in each fraction from 1 to 30 were determined by NTA and the absorbance at a wavelength of 280 nm, respectively. Fractions 1–5 were subsequently pooled together as EV samples (blue dotted line).

We have optimized the protocol of EV isolation using SEC (Figure 1). The starting volume of platelet-free plasma was 0.25 ml and added to 1.75 ml of PBS, resulting in an eightfold dilution. The initial volume of 2 ml was subjected to SEC, and a total of 30 fractions of 1 ml were collected (Figure 1A). As the larger molecules elute first, followed by smaller protein complexes (Figure 1A), EVs eluted first from SEC. The particle number of EVs in each fraction was determined by NTA. Based on NTA data, fractions 1–5 were pooled as EV samples for higher purity (Figure 1B). Indicative protein elution profiles can be obtained by monitoring the absorbance at a wavelength of 280 nm. It is critical to remove abundant plasma proteins from EV samples to improve the purity and we found that soluble protein elution rapidly increases from fraction 7 (Figure 1B). Then, EV-enriched samples from fractions 1–5 were further analyzed.

### Physical and Biochemical Characterization of Extracellular Vesicles

**FIGURE 2:**
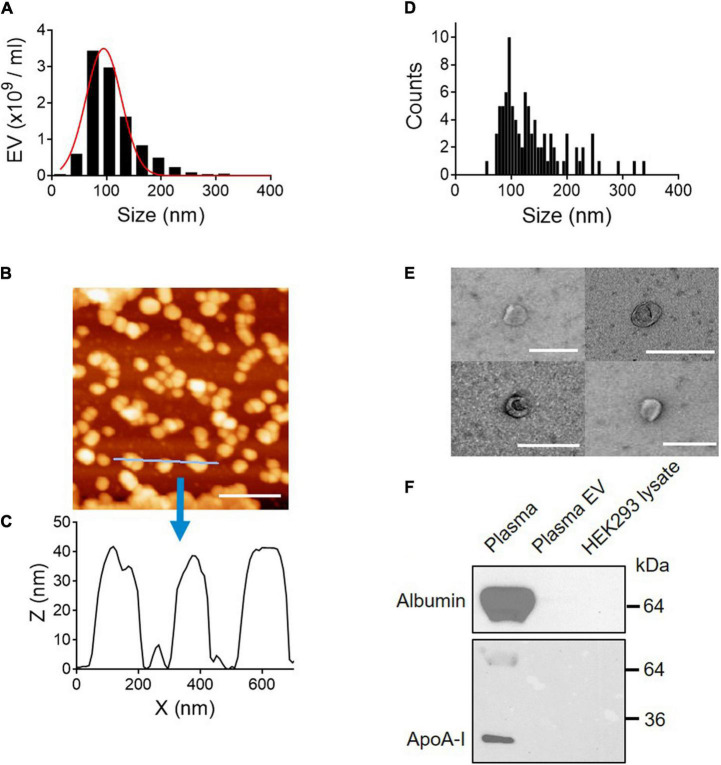
Physical and biochemical characterization of EVs isolated from plasma. **(A)** Size distribution of plasma EVs determined by NTA. Median diameter (nm), 90.2 ± 4.58 SD. **(B)** AFM image (scale bar, 400 nm) and **(C)** line scan profiles of isolated EVs. **(D)** Size distribution of plasma EVs determined by AFM. **(E)** Negative-stain TEM images of EVs. Scale bar, 200 nm. **(F)** Purity of EV samples tested by Western blot. Plasma proteins, albumin, and ApoA-I are removed from plasma EV samples. Seven micrograms of protein from whole plasma, plasma EVs isolated using SEC, and HEK293 cell lysate were subject to SDS–PAGE and immunoblotted with antibodies against albumin and ApoA-I.

The size distribution of plasma EVs was determined by NTA techniques, which use dynamic light scattering (DLS) and Brownian motion to accurately identify the size and quantity of EVs in a suspension (Dragovic et al., 2015). The size of isolated plasma EVs mainly ranges from 50 to 150 nm with 90.2 ± 4.58 SD of median diameter (nm) (Figure 2A). Furthermore, we characterized the 3D structure of EVs using AFM (Figures 2B–D). The AFM technique allows to obtain an actual 3D image of surface topography with very high resolution and measure EV samples in native conditions without chemical fixation that might cause artifact during sample preparation. The EV height in the *z*-axis direction is around 40 nm (Figure 2C) and the size distribution of EVs determined by AFM is from 50 to 200 nm in diameter; median diameter (nm): 110 ± 37.3 SD (Figure 2D), correlating with the NTA data (Figure 2A). The height of EVs is smaller than the diameter (Figures 2C,D), because EVs become flattened after binding to the mica surface and the height/diameter ratio of EVs is much less than 1 (Vorselen et al., 2020).

Next, the membrane structure of negatively stained plasma EVs was further characterized by transmission electron microscopy (TEM). TEM images show EVs surrounded by a lipid membrane, and the size of EVs is around 100 nm in diameter (Figure 2E), which is in line with the NTA and AFM data. To further validate the EV purity, the presence of albumin and ApoA-I, a component of high-density lipoprotein (HDL), was assessed by Western blot analysis. Albumin is the most abundant plasma protein (Belinskaia et al., 2021), and lipoproteins are co-isolated with plasma EVs as major contaminants (Brennan et al., 2020). Both albumin and ApoA-I, abundant in plasma, were removed entirely from EV samples (Figure 2F). Taken together, EV samples isolated using SEC are free of plasma proteins and small in size, from 50 to 150 nm in diameter.

### Extracellular Vesicle Characterization by Flow Cytometry

**FIGURE 3:**
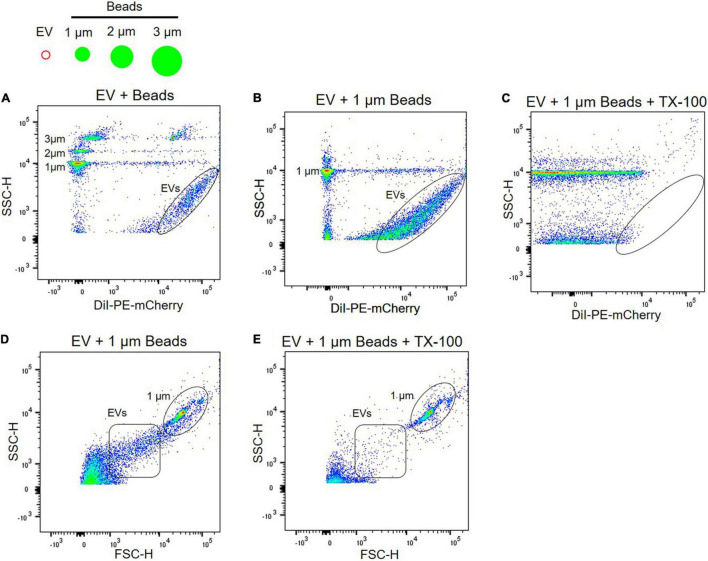
Characterization of EVs using flow cytometry. **(A)** Plasma EVs labeled with DiI were compared with different sizes of beads, i.e., 1, 2, and 3 μm. Light scatter signals correspond to the size of beads, and DiI labeling of EV samples (black circle) increases fluorescence signal. Plasma EVs labeled with DiI (black circle) were mixed with 1 μm beads in the absence **(B)** and presence **(C)** of 0.1% Triton X-100. Light scatter and fluorescence signals of DiI-labeled EVs were absent after detergent lysis. Dot plot of forward scatter (FSC) signal for plasma EVs (black square) and 1 μm beads (black circle) in the absence **(D)** and presence **(E)** of 0.1% Triton X-100.

Due to the heterogeneity of EV populations, sensitive and reproducible methods for single EV analysis are critical to investigate plasma EVs as biomarkers and therapeutic targets for neurological disorder (Welsh et al., 2020). Flow cytometry is the key technology for analyzing individual EVs, but most commercial flow cytometers are designed for cell analysis, two orders of magnitude larger than EVs. Because plasma EVs are small, from 50 to 150 nm in diameter (Figure 2), standard flow cytometers have limitations in the instrument sensitivity. They might contain artifacts caused by insufficient instrument sensitivity and inappropriate calibration (Welsh et al., 2020). To address the issue of the instrument sensitivity to analyze individual plasma EVs, we used high-sensitivity flow cytometry, BD FACS Aria III SORP, which is suitable and sensitive enough to detect small EVs with diameters below 200 nm (Gorgens et al., 2019). Plasma EVs were labeled with DiI dye, a fluorescent lipophilic cationic indocarbocyanine dye that diffuses laterally to stain the entire membrane organelles. Polystyrene beads of defined sizes with 1–3 μm were used to standardize flow cytometers and compare with plasma EVs (Figure 3). DiI-labeled EVs are heterogeneous in size and around one order of magnitude smaller than 1 μm beads (Figures 3A,B), again correlating with the size distribution of EVs determined by NTA and AFM (Figures 2A,D).

Next, we further confirmed if light scatter signals in flow cytometry originate from protein complexes or membrane-enclosed vesicles using detergent, 0.1% Triton X-100. Detergent lyses membrane-enclosed vesicles, thus reducing signals, whereas detergent-resistant particles, including protein complexes or HDL, remain stable following detergent treatment. Detergent control is useful for determining whether detected events represent membrane-enclosed vesicles or protein complexes (Welsh et al., 2020). EVs were labeled with DiI and almost all scatter signals and events of DiI-labeled EVs were absent after detergent lysis; scatter signals of 1 μm beads persist in detergent (Figures 3B,C). Forward scatter signals of plasma EVs were also disrupted following detergent lysis (Figures 3D,E).

**FIGURE 4:**
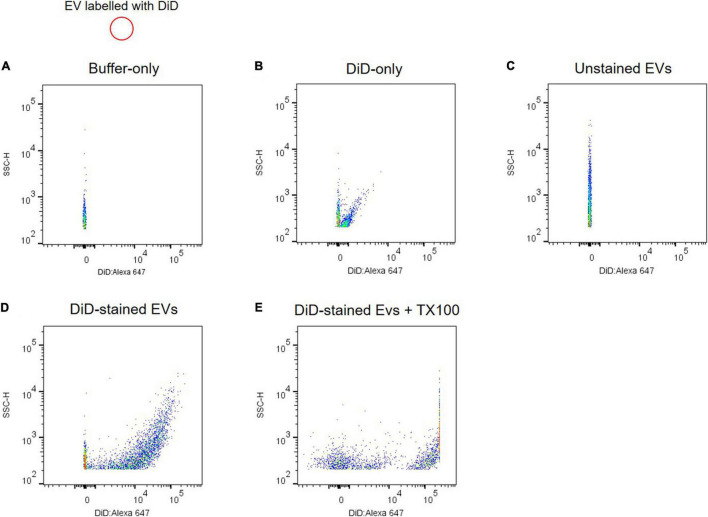
Analyzing individual EVs using flow cytometry. Shown are dot plots of fluorescent intensity for plasma EVs labeled with DiD. Background acquisition with buffer-only control **(A)** and DiD-only control **(B)**. Plasma EV samples were unstained **(C)** and stained with DiD (black circle) **(D)**. **(E)** 0.1% Triton X-100 treatment disrupts light scatter and fluorescent signals of DiD-labeled EVs.

Due to artifacts and technical challenges in monitoring submicron EVs with 50–100 nm in diameter using flow cytometry, several controls are required to validate scatter signals of plasma EVs. A buffer-only control provides background and a reference of the recorded event rate at the same settings used to analyze plasma EV samples (Figure 4A). Plasma EVs were labeled with DiD, a fluorescent lipophilic dye, and DiD-only control showed fluorescence-positive events and light scatter in the absence of plasma EV samples (Figure 4B). DiD-only control is important to validate EV’s positive signals because aggregates of DiD dye may be detected as the signal and artifactually interpreted as EVs (Morales-Kastresana et al., 2017; Welsh et al., 2020). Unstained EV control is useful to compare with DiD-labeled EVs for the light scatter signal intensity (Figure 4C). DiD-labeled EVs led to the increase of fluorescence-positive signal events (Figure 4D), which were absent after Triton X-100 lysis (Figure 4E). Altogether, these data provide evidence that plasma EVs are membrane-enclosed vesicles without protein complexes, and individual plasma EVs can be analyzed using flow cytometry.

### Analyzing Individual Extracellular Vesicles Using Flow Cytometry

**FIGURE 5:**
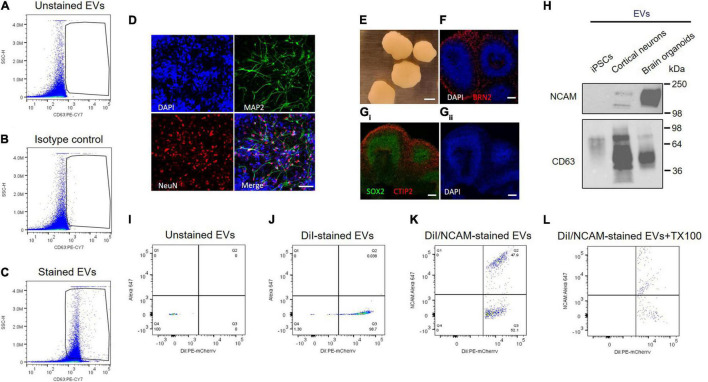
NCAM as a neuronal exosome marker in plasma. **(A–C)** Quantitative analysis of plasma EVs using flow cytometry. Dot plots of fluorescent intensity for plasma EVs stained with PE-CY7-labeled CD63 antibody. EVs were unstained **(A)** and stained with either isotype control **(B)** or CD63 antibody **(C)**. **(D)** A18945 iPSC-derived neurons cultured for 6 weeks and immunostaining with mature neuronal markers (DAPI, MAP2, NeuN, and merged image). Images were taken using Zeiss LSM confocal microscope at ×63 magnification. Scale bar, 20 μm. **(E)** Representative images of cortical organoids at day 90 of differentiation. Scale bar, 1 mm. **(F)** The organoids express cortical layer marker BRN2 (also called POU3F2). The nuclei were stained with DAPI. Scale bar, 100 μm. **(G_i_)** Markers for proliferating neural progenitors (SOX2) and cortical neuron marker CTIP2 (also known as BCL11B) with nuclei DAPI staining **(G_ii_)**. **(H)** Western blot analysis of NCAM and CD63 from EVs released from iPSCs, iPSC-derived cortical neurons, and iPSC-derived brain organoids. EVs were isolated using SEC from the cell culture media of each sample, and equal EV particle numbers (6 × 10^8^) were subject to immunoblotting with NCAM and CD63 antibodies. **(I–L)** Flow cytometry dot plots of fluorescent intensity for plasma EVs double-stained with DiI and NCAM antibody. EV samples were unstained **(I)**, single stained with DiI **(J)**, and double-stained with DiI and NCAM antibody in the absence **(K)** and presence **(L)** of 0.1% Triton X-100.

Individual EVs isolated from plasma were tested using an antibody against CD63, a selective exosome marker. Several tetraspanins, including CD9, CD63, and CD81, have been used for markers of EVs (Kowal et al., 2016), but CD63 is a reliable marker for endosome-derived exosomes, whereas CD9 and CD81 are enriched in ectosomes, which bud from the plasma membrane (Mathieu et al., 2021). EVs were stained with PE-labeled anti-CD63 antibody, and > 95% of plasma EVs we isolated here are CD63 positive, whereas unstained EV control is almost identical with isotype control (Figures 5A–C). Isotype control compared to unstained EV control can provide a valuable method to identify antibody specificity and false-positive signals. Our flow cytometry data support that plasma EVs isolated here are mostly exosomes.

Next, we tested NCAM as a marker for neuronal EVs using human iPSCs-derived cortical neurons and brain organoids (Figures 5D–H). Human iPSC-derived cortical neurons express neuron-specific markers including microtubule-associated protein 2 (MAP2) enriched in dendrites and neuronal nuclear protein (NeuN), confirming neuronal maturity (Figure 5D). Human iPSC-derived brain organoids also express cortical neuron layer marker proteins such as BRN2, also called POU3F2, and CTIP2 (Figures 5E–G). SOX2 is expressed in neural stem cells and proliferating neural progenitors and we observed that inner layers of brain organoids are mainly SOX2-positive neural progenitors, whereas outer layers express CTIP2, a mature cortical neuron marker (Figure 5G_i_).

First of all, NCAM expression was compared between EVs released from either iPSCs, iPSC-derived cortical neurons, and iPSC-derived brain organoids (Figure 5H). Interestingly, NCAM is highly enriched in EVs released from iPSC-derived cortical neurons and iPSC-derived brain organoids, but not in EVs secreted from iPSCs, which are non-neuronal cells, supporting that NCAM can be a good marker for neuronal EVs (Figure 5H). Finally, to investigate NCAM-positive individual EVs, we performed double stainings. EV samples were simultaneously stained with DiI, a fluorescent lipophilic dye, and an anti-NCAM antibody (Figures 5I–L). We detected DiI/NCAM double-positive events from plasma EVs (Figure 5K), completely disrupted following detergent lysis (Figure 5L). Taken together, plasma EVs, mostly exosomes, contain NCAM-positive EVs, which might be neuronal EVs and can be used to decipher the respective functions of neuronal EVs and biomarker discovery for neurological disorders.

## Discussion

Prognosis and diagnosis of neurological disorders and brain diseases mainly depend on behavior and postmortem analysis, when conditions have already manifested. It is still challenging to study pathological and biochemical processes of neurological and psychiatric disorders because access to neurons and other brain cells in living human individuals is limited. Blood plasma has the advantage of being non-invasive and easy access to the liquid biopsy (Marrugo-Ramirez et al., 2018). Because EVs secreted from dysregulated cells and tissue reflect disease severity, the contents of EVs have a potential clinical utility as novel biomarkers for early diagnosis and therapeutic targets for treatment (Trino et al., 2021). EVs can cross the BBB (Alvarez-Erviti et al., 2011; Chen et al., 2016; Saeedi et al., 2019), circulating through the bloodstream. Therefore, plasma EVs provide a platform for a liquid biopsy to diagnose neurological disorders.

However, all cell types release exosomes and EVs into the blood circulation, and it is challenging to characterize neuron-specific EVs isolated from plasma for the early diagnosis of neurological disorders. NCAM and L1CAM have been suggested as neuronal EV markers, however, a recent study demonstrated that L1CAM is found mainly in soluble fractions of plasma and not associated with EVs (Mustapic et al., 2017; Norman et al., 2021), arguing against L1CAM as a neuronal EV marker. Markers for neuron-derived EVs remain controversial and under debate. Here our data show that NCAM can be a good marker for neuronal EVs using single EV analysis and brain organoids (Figure 5), suggesting that NCAM-positive neuronal EVs from plasma have potential as a means of direct access to pathology and biomarkers for early diagnosis of neurological disorders.

Analysis of EVs using conventional flow cytometry has its limitations. Unlike the cells that are in micrometer range, flow cytometers have a limited capacity to detect nanoparticles below 200 nm. The overlapping background noise from the buffers, optics, and electronics is a major problem that needs to be tackled. A broadly used method is to couple EVs to beads which results in larger particles and reliable detection on the conventional flow cytometer. However, bead-based flow cytometry has limited utility because of restriction in the discrimination between EVs and contaminating protein aggregates present in the isolated sample; e.g., it is impossible to discriminate EV-associated L1CAM from soluble L1CAM using bead-based flow cytometry. The beads approach is inconvenient for subsequent use of EVs and is highly dependent on antigen abundance and antibodies quality. Another challenge is that EVs are dim particles with a low refractive index, which hinders the use of standard latex beads for quality control and instrument standardization. To overcome some of these challenges, it was critical to use only 0.22 μm filtered PBS buffer and to acquire the events at the lowest flow rate, which significantly reduces the background noise and enhances the instrument sensitivity as the particles slowly pass through the laser intercepts.

Most studies use bulk analyses of heterogeneous EVs using Western blot and ELISA without quantitative data. We provide evidence that neuronal EVs released from human iPSC-derived cortical neurons and brain organoids enrich NCAM, suggesting that NCAM is a promising neuronal EV marker. Flow cytometry analysis of individual plasma EVs gives rise to quantitative data of NCAM-positive plasma EVs, which might be used for biomarker discovery of neurological disorders as candidate neuronal EVs. NCAM, also called CD56, is highly expressed in neurons, but also present at low levels in lymphocytes in blood (Van Acker et al., 2017). Therefore, it remains a topic of further study if NCAM-positive plasma EVs mainly originate from the brain and can be targeted for biomarker discovery of neurological and neurodevelopmental disorders, including autism spectrum disorder.

## Data Availability Statement

The raw data supporting the conclusions of this article will be made available by the authors, without undue reservation.

## Ethics Statement

This study was approved by the Institutional Review Board (IRB# 2018-024) of Qatar Biomedical Research Institute (QBRI). The patients/participants provided their written informed consent to participate in this study.

## Author Contributions

HAM and KS purified EVs. HAM, NM, GA, SMaa, SL, VG, YT, JP, and SS-A performed the experiments. FA-S collected blood samples. SMan, H-GK, LS, J-CG, SA, AA-S, and YP collected and analyzed the data. HAM and YP wrote the manuscript. All authors read and provided their comments.

## Conflict of Interest

The authors declare that the research was conducted in the absence of any commercial or financial relationships that could be construed as a potential conflict of interest.

## Publisher’s Note

All claims expressed in this article are solely those of the authors and do not necessarily represent those of their affiliated organizations, or those of the publisher, the editors and the reviewers. Any product that may be evaluated in this article, or claim that may be made by its manufacturer, is not guaranteed or endorsed by the publisher.
